# A critical analysis of health care purchasing arrangements in Kenya: A case study of the county departments of health

**DOI:** 10.1002/hpm.2604

**Published:** 2018-08-03

**Authors:** Rahab Mbau, Edwine Barasa, Kenneth Munge, Stephen Mulupi, Peter K. Nguhiu, Jane Chuma

**Affiliations:** ^1^ Health Economics Research Unit KEMRI Wellcome Trust Research Programme Nairobi Kenya; ^2^ Nuffield Department of Medicine Oxford University Oxford UK; ^3^ The World Bank Kenya Country Office Nairobi Kenya

**Keywords:** county departments of health, Kenya, strategic purchasing, universal health coverage

## Abstract

**Background:**

Purchasing in health care financing refers to the transfer of pooled funds to health care providers for the provision of health care services. There is limited empirical work on purchasing arrangements and what is required for strategic purchasing in low‐ and middle‐income countries. We conducted this study to critically assess the purchasing arrangements of the county departments of health (CDOH) who are the largest purchasers of health care in Kenya.

**Methods:**

We used a qualitative case study approach to assess the extent to which the purchasing actions of the CDOH in Kenya were strategic. We purposively sampled 10 counties and collected data using in‐depth interviews (n = 81), focus group discussions (n = 4), and documents review. We analyzed data using a framework approach.

**Results:**

County departments of health did not practice strategic purchasing. The government's (national and county) role as a steward for the purchasing function was characterized by poor accountability and inadequate budgetary allocations for service delivery. The absence of a purchaser‐provider split between the CDOH and public health care providers undermined provider selection based on performance and quality. Poor public participation and ineffective complaints and feedback mechanisms limited public accountability and responsiveness to the needs of the people.

**Conclusion:**

Our findings show that while there are frameworks that could promote strategic purchasing of the CDOH, strategic purchasing is impaired by poor implementation of these frameworks and the inherent weaknesses of a public integrated purchasing system that lacks purchaser‐provider split.

## 1 INTRODUCTION

Universal health coverage (UHC), which refers to access to needed health services that are of good quality without financial hardship,^1^ requires well‐functioning health financing systems.^2^ There are three interrelated health financing functions: (1) revenue collection—a process by which funds are collected from individuals or households, organizations, governments, and donors; (2) pooling of funds—accumulation of revenue on behalf of a population; and (3) purchasing—the transfer of the pooled resources to health care providers for the provision of health services.^3^, ^4^ Purchasing of health care services, as a function of health care financing, is distinguished here from the procurement of medical commodities such as medicines and medical equipment. Many countries are implementing health financing reforms towards attaining UHC.^2^, ^5^, ^6^, ^7^ These reforms typically focus on the revenue collection and pooling functions of a health system, with less attention directed towards the purchasing function.^2^

Purchasing provides a critical link between revenue collection and the provision of health services.^8^ This is because purchasing involves three key decisions, namely, (1) which interventions to purchase using available resources, (2) from who (service providers), and (3) how (provider payment mechanisms).^3^, ^9^ These decisions can be made either passively or strategically. Passive purchasing involves the transfer of funds to health care providers based on historical or predetermined budgets without consideration of efficiency. Strategic purchasing involves transfer of funds that incentivizes health care providers to seek equity, efficiency, and quality in service delivery,^8^, ^10^ which leads to responsiveness, improved health outcomes, and financial risk protection.^3^ Purchasing arrangements fall under either of two broad models.^11^ The first model is a contract system where the purchaser is organizationally separate from health service providers.^11^ For example, a social insurer entering into contracts with public and private health care providers to provide health care services to the social insurer's members is operating under a public contract system. A private insurer using a similar arrangement is operating under a private contract system. The second model is the integrated system where the purchaser and the provider belong to the same organization hence no purchaser‐provider split.^11^ For example, a country's ministry of health (MOH) could purchase health care services from public health care providers that it owns and hence operate under a public integrated purchasing system. A health management organization that purchases health care services from private providers that it owns operates under a private integrated system.

Purchasing in Kenya is done under both models. Under the contract model, the National Hospital Insurance Fund purchases services from public and private health care facilities that it contracts (public contract), while private health insurers (including community‐based health insurers and micro–health insurers) purchase health care services from public and private providers that they contract (private contract). Under the integrated model, the national Ministry of Health (MOH) purchases services from public tertiary hospitals that it owns (public integrated), while the county departments of health (CDOH) purchase services from public secondary care hospitals and primary health care facilities that they own (public integrated). In the financial years 2015 to 2016, the national government allocated 59 billion Kenya shillings (590 million USD) to the national MOH, while county governments allocated Kenya shillings 85 billion (USD 850 million) to the CDOH.^12^ Financial resources controlled by the National Hospital Insurance Fund and private health insurers, as a proportion of total financial resources available for the health sector, are typically much lower than those controlled by the national MOH and CDOH.^13^ County departments of health are therefore the largest health care purchasers in Kenya.

Given the CDOH's key role in purchasing health care services in Kenya, understanding their purchasing arrangements is critical. This study aims to examine the extent to which the purchasing actions of the CDOH are strategic along each of the three CDOH purchasing relationships (Government‐CDOH, CDOH‐health care providers, and citizen‐CDOH) based on a theoretical framework of ideal strategic purchasing actions (SPAs) that was developed by the Resilient and Responsive Health Systems (RESYST)^8^ consortium.

## 2 METHODS

### 2.1 Study setting

Kenya has a devolved system of governance with two distinct but interdependent governments: one national government and 47 county governments.^14^ The national government formulates national health policies and oversees national referral hospitals through the MOH. The county governments implement national health policies and oversee health service provision at the county level through the CDOH.^14^, ^15^ Preliminary estimates of the 2015/2016 National Health Accounts show that the health sector is financed from three major sources: the government, households, and donors, which account for 40%, 31%, and 29% of the total health expenditure, respectively. Health services in Kenya are provided by both public and private health care providers in an almost equal share.^16^ The provision of health care services in the public sector is organized into four tiers: tier 1, community health units; tier 2, primary care (dispensaries and health centers); tier 3, secondary care (county hospitals); and tier 4, tertiary care (national referral hospitals).^15^

### 2.2 Study design

We used a qualitative case study approach given its appropriateness in empirical enquiry of a complex phenomenon in real‐life context^17^ as is the purchasing arrangement. The CDOH purchasing arrangements formed the “case” in this study.

### 2.3 County selection

We selected 10 study counties purposively taking into consideration variability in population size, sociodemographic and economic factors, and health indicators. We also considered logistical feasibility of data collection in the selected counties with regards to safety and security for the study team members. Table 1 outlines the characteristics of selected counties. For ethical reasons, we have anonymized the counties to protect the confidentiality of study participants. K.M. and S.M. made introductory calls to the selected study counties to gain institutional approval for the study.

**Table 1:** Characteristics of the selected study counties

County	Projected Population 2015^a^	Urban Population^a^	Poverty Rate^a^	Immunization Coverage^a^	Percentage of County Budget Allocated to Health (2015/2016)
A	1 119 769	8%	40%	68.5%	22%
B	1 336 590	21.9%	71.7%	78.7%	21%
C	577 783	9.8%	36%	98.3%	26%
D	783 261	17.6%	74.9%	77%	20%
E	1 271 920	20%	38%	85%	12%
F	1 013 325	10.7%	36.3%	92%	19%
G	722 498	4%	46.3%	86%	12%
H	832 877	16%	28.8%	88.3%	41%
I	902 753	5.6%	47.6%	86%	31%
J	297 579	25.5%	57.2%	70.9%	26%

a County specific–integrated development plans.

### 2.4 Data collection

We collected data between August and November 2015 through in‐depth interviews (IDIs), focus group discussions (FGDs), and document reviews. Prior to IDIs and FGDs, each participant received a research information sheet with information on the purpose of the study, their rights, time involvement, and potential risks and benefits. Written informed consent was then obtained with permission from the participants. The researchers used interview topic guides for the IDIs and FGDs. These topic guides were informed by the study's objectives and conceptual framework. To enhance rigor (validity and trustworthiness), draft topic guides were presented and extensively discussed with a group of experienced researchers to explore the extent to which they appropriately reflected the concept of strategic purchasing (construct validity) and comprehensively captured all the dimensions of the subject of study (content validity). The tools were then revised based on inputs from this process.

K.M., S.M., and P.N. conducted IDIs with 81 participants (Table 2) who were selected purposively (based on their knowledge on health financing and purchasing arrangements within the county government) and through snowballing. Each IDI was audio recorded in English and lasted between 30 and 45 minutes. A total of 20 FGDs were conducted with members of the public who were selected on the basis of age and gender to enable meaningful interactive discussions devoid of intimidation (n = 9 men; 7 women; 1 mixed; and 3 civil society organizations). Each FGD was audio recorded and lasted between 60 and 90 minutes. Eleven FGDs were not in English (n = 10 were in Swahili, and n = 1 was in Kikuyu) and were translated into English.

**Table 2:** In‐depth interview participants included in the study

County Respondents		
	Cadre	Number
Senior‐level managers	County director of health	7
	Chief officer of health	6
	Senior economist	5
	County budget coordinator	5
	Member of the county assembly	5
	Chief officer of finance and budgeting	2
	Chief officer of supply chain management	1
Middle‐level managers	County pharmacist	8
	County health records information officer	5
	County community health focal person	5
	County nursing officer in‐charge	4
	County public health officer	4
	Human resource manager, county public service board	1
	Total	58
Facility respondents		
Facility‐level managers	Medical superintendent	9
	Nursing officer in‐charge	5
	Hospital administration officer	7
	Hospital records information officer	2
	Total	23

The researchers reviewed national and county‐level statutory and regulatory documents and budget reports that were perceived to be relevant to the purchasing function (Table 3).

**Table 3:** Documents reviewed in the study

National Statutory Documents	The Constitution of Kenya
	The Kenya Health Policy 2014‐2030
	Kenya Health Sector Strategic and Investment Plan (KHSSP)
	Kenya Health Sector Referral Strategy
	Kenya Essential Package for Health
	Kenya National e‐Health Strategy 2011‐2017
	National and county health budget analysis reports
	Public Finance Management Act
	Vision 2030
County Statutory Documents	County Governments Act
	County executive budget
	County finance acts
	County integrated development plans

### 2.5 Conceptual framework

**Figure 1:**
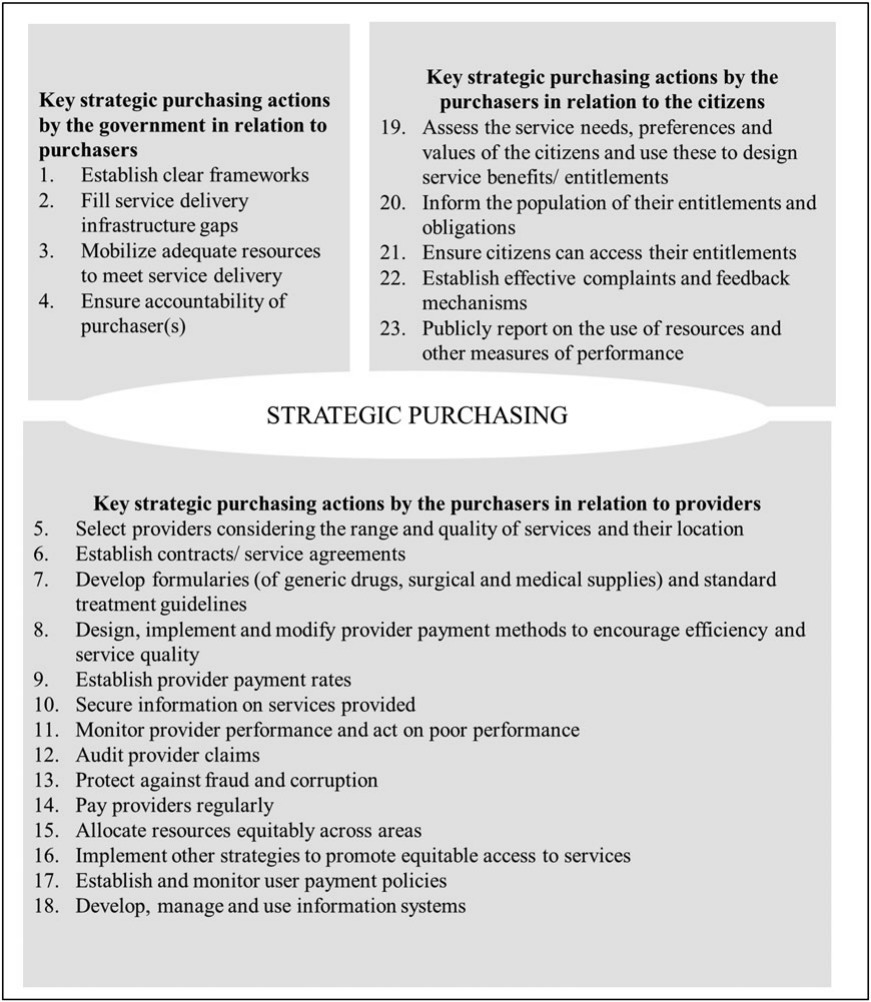
RESYST framework on ideal strategic purchasing actions

This study was guided by the Figueras et al^9^ and the RESYST^8^ frameworks. According to the Figueras et al framework, purchasing is characterized by relationships between the purchaser and three key actors: the government, health care providers, and citizens.^9^ Using the Figueras et al framework, we identified three purchaser‐relationships between (1) the CDOH and the government (both county and national), (2) the CDOH and health care providers, and (3) the CDOH and the citizens. We combined the county and national government because under Kenya's devolved system, the national government does not interact directly with county departments, but rather through the county governments. We then compared the actual purchasing practices conducted under each of the three purchaser‐relationships to the ideal SPAs outlined in the RESYST framework (Figure 1). The RESYST framework is a theoretical framework that outlines 23 SPAs drawn from available literature on strategic purchasing and from the experiences and understanding of the RESYST consortium members.^8^

### 2.6 Data analysis

All IDIs and FGDs were transcribed and imported into QSR NVIVO 10 software to manage coding and analysis.^18^ K.M., P.N., and R.M. back‐checked the transcripts with the audios to ensure transcription and translation accuracy. We then analyzed the data deductively using a framework approach that involves familiarization with the data; identification of a thematic framework; data coding; charting; and, lastly, mapping and interpretation.^19^ We familiarized ourselves with the data through reading and rereading of all textual data (transcripts and policy documents) and listening to audio recordings. We then developed a thematic framework based on the study's conceptual framework and the themes and ideas emerging from the familiarization process. We then independently coded the data by applying the developed thematic framework to all the data and compared the codes for intercoder variability. Where there were variations, we consulted each other and agreed on the codes to apply. We identified emerging themes and charted the data to these themes by lifting the data from their original contexts and laying them out into themes. These themes were then supported by quotes from IDIs and FGDs that were most illustrative of the theme.^20^ We completed the analysis by interpreting the data as a whole based on associations that were relevant to the objectives of the research study.

### 2.7 Ethics

We received ethical approval from the KEMRI scientific and ethics research unit under number 2795. We also obtained approval from the relevant counties and health facilities followed by written informed consents from all the participants who agreed to participate in the study.

## 3 RESULTS

In this section, we present findings of the analysis of purchasing arrangements of the CDOH under each of the three purchaser relationships. Under each of these relationships, we present findings on the extent to which the purchasing actions were strategic (or not), and where applicable, we link our findings and the RESYST framework by indicating the relevant SPA number in parenthesis.

### 3.1 Government—CDOH relationship

#### 3.1.1 Existence of policies and legal frameworks that guided purchasing by the CDOH

An SPA by governments in their relationship with purchasers is the establishment of clear frameworks in the form of policies and legislation that guide purchasing (SPA 1). Such policies and legislation existed in the relationship between the government (national and county) and the CDOH. Specifically, the CDOH's decisions on which services should be purchased, from whom and how were informed by the Constitution of Kenya, acts of parliament and national health policies. One, services to be purchased were outlined in the Kenya Essential Package for Health (KEPH) in line with the Kenya Health Policy 2014 to 2030 15 and the 2013 to 2017 Kenya Health Sector Strategic and Investment Plan.^21^ The KEPH is composed of promotive, preventive, curative, and rehabilitative health services, differentiated according to the tier of care.

We have a Kenya Essential Package for Health which basically outlines what services ideally are supposed to be at the health center level, the dispensary level, the community level. So that is our guiding policy. Senior‐level manager, County C

Two, the CDOH purchased health services from tier 1 (community health units), tier 2 (health centres and dispensaries), and tier 3 (county hospitals) public health care providers within their jurisdiction.^14^, ^15^ Health services purchased from these providers were expected to contribute towards the attainment of medium and long‐term indicators and targets for health service outcomes in the health sector as outlined in the national health policies.^15^, ^22^ These national health policies (the KEPH, Kenya Health Policy, and Kenya Health Sector Strategic and Investment Plan) were embedded in the broader national policy‐Kenya's Vision 2030—which aimed to achieve UHC^23^ by 2030. The county governments adopted these health policies in the development of the County Integrated Development Plans (CIDP), which are 5‐year policy frameworks for the county. The CIDP informed annual priority setting of health service delivery and investment through annual development plans and budgets for the CDOH and other departments.

Whatever we plan at the county level should be in line with what the national government is targeting like vision 2030. We cannot be going left and the national government going right. Senior‐level manager, County J

Third, decisions on how to pay for the services were informed by the Public Finance Management Act (PFMA)^24^ and related regulations. These regulations specified the allocation of budgets for service delivery and informed financial management processes including funds flow and use between the county governments and public health care providers.

The budgeting cycle is spearheaded by the county treasury but we, as the department of health, take part in that. The treasury tries as much as possible to ensure they are working within the public finance management act. The treasury gives ceilings to the departments. We put in budgets for the hospitals and once they are approved by the county assembly, then the funds start flowing in. Senior manager, County I

#### 3.1.2 Inadequate service delivery infrastructure

Governments are expected to fill service delivery infrastructure gaps (SPA 2). This SPA was not met since there was inadequate service delivery infrastructure. The norms and standards for health workers and health facilities (by population and by distance) were outlined in the KEPH. These norms were based on Kenya's burden of disease.^25^ Tables 4 and 5 compare the actual number of public health workers (doctors and nurses) and public health facilities in the 10 study counties to the KEPH norms. It is clear that there are infrastructure gaps in most of these counties. These were blamed on budget constraints and poor prioritization during the budget making process.

We need to prioritize our investments. We are very busy buying ambulances yet we do not have staff. His Excellency has also given us digital X‐ray machines. We have more than eight machines but they are being operated by only two persons. The number of patients that are waiting is overwhelming. Facility‐level manager, County B

**Table 4:** Human resource for health, required, and actual numbers

County	Required Doctors^a^ (4.2 per 10 000 Population)^25^ Based on 2015 Population	Actual Number of Doctors Based on 2015 Population^26^	Percentage Gap	Required Nurses^b^ (8.1 per 10 000 Population)^25^ Based on 2015 Population	Actual Number of Nurses Based on 2015 Population^26^	Percentage Gap
A	470	34	92.9%	907	448	50.6%
B	561	53	90.5%	1,083	400	63%
C	242	29	88.2%	468	502	…
D	329	31	90.3%	634	274	56.8%
E	534	661	…	1,030	1679	…
F	426	30	93%	821	304	63%
G	303	58	80.9%	585	441	24.6%
H	350	92	73.7%	675	533	21%
I	379	18	95.3%	731	298	59.2%
J	125	42	66.4%	241	205	14.9%

a Doctors (medical officers and specialists).

b Nursing staff (Bachelor of Science in Nursing, Kenya Registered Community Health Nurse, and Kenya Enrolled Community Health Nurse).

**Table 5:** County health facilities, required, and actual numbers

County	Required Number of Public Health Facilities in the County based on KEPH Health Facility Norms using the 2015 Population (One Dispensary [Tier 2] per 10 000 Population, one Health Center [Tier 2] per 30 000 Population, and one Hospital [Tier 3] per 100 000 Population)^25^	Actual Number of Public Health Facilities in the County^26^	% Gap
A	161	147	8.7%
B	192	107	44%
C	83	60	27.7%
D	112	74	33.9%
E	183	58	68.3%
F	145	119	17.9%
G	104	65	37.5%
H	119	120	…
I	129	123	4.7%
J	43	62	…

Abbreviation: KEPH, Kenya Essential Package of Health.

The public health facilities also lacked essential health commodities such as pharmaceuticals and non‐pharmaceuticals that were required for service delivery. Document reviews showed that health products particularly for noncommunicable disease, maternal health services, and pediatrics were not readily available.^16^

When you come on the ground, you find there are no drugs, non‐pharmaceuticals, cleaning products, linen. We cannot work without these commodities. Facility level manager, County C

#### 3.1.3 Inadequate allocation and unpredictable disbursement of financial resources for service delivery

**Figure 2:**
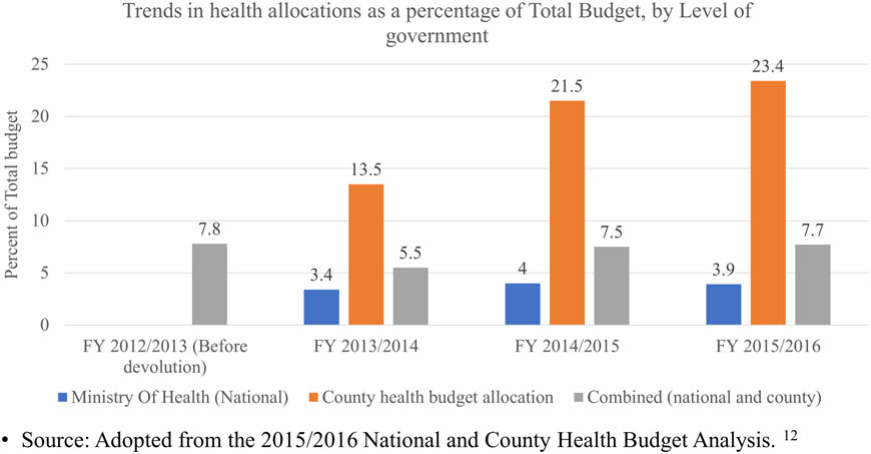
Health budget allocations by level of government (national and all the 47 county governments) [Colour figure can be viewed at wileyonlinelibrary.com]

Governments are expected to mobilize adequate resources to meet service delivery (SPA 3). This SPA was not met for various reasons. One, combined allocation to the health sector by both the national and county governments was below the 15% target recommended by the Abuja declaration^12^ (Figure 2). While county budget allocations to the health sector exceeded the 15% target, the absolute revenue was low due to the low national budget allocations to health. In fact, the general government expenditure on health as a percentage of Kenya's gross domestic product^26^ was 2.3% in 2013, which is below the 5% threshold that has been recommended for low‐ and middle‐income countries.^27^

Two, in practice, county health budget allocations were associated with budget cuts and reallocations to other departments, which limited resources available for service delivery.

But now due to budget cuts, reallocation to other departments, the budget was actually cut to two thirds of what we had initially proposed. Senior‐level manager, County G

**Figure 3:**
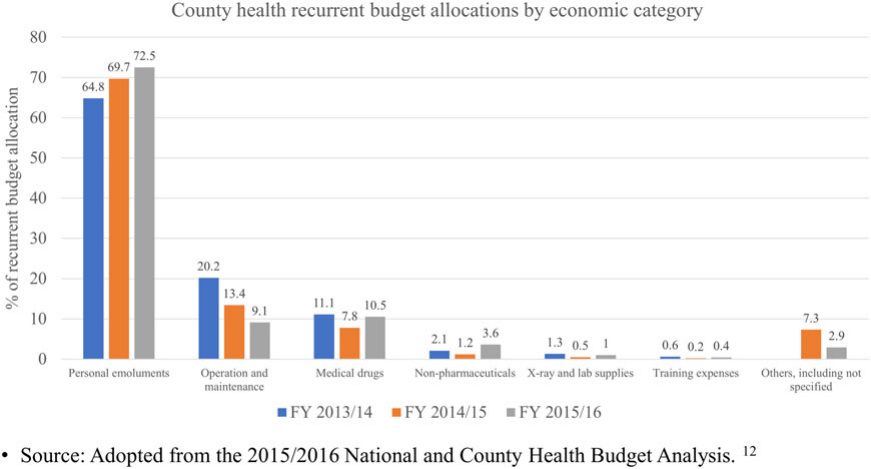
Recurrent health budget allocations by economic category in all the 47 county governments [Colour figure can be viewed at wileyonlinelibrary.com]

Three, inadequate allocation of funds for service delivery also occurred during reallocation of the county health budgets into recurrent and development budgets. According to the PFMA, recurrent and development expenditure should account for 70% and 30% (at least) of the health budget, respectively. While the recurrent share looks high, in practice, about 70% of this budget went into personnel emoluments leaving very little money for pharmaceuticals, non‐pharmaceuticals, and other medical supplies^12^ that are required for service delivery (Figure 3).

Lastly, delays and unpredictability in the disbursement of resources from the county treasury to the CDOH undermined the availability of resources for service delivery. These delays could be attributed in part to delays in disbursements from the national government to the county treasury.

We have planned for the year 2015/2016. Half the year is gone; we have not received any funding. So, there is that disconnect between what we had planned, what we had budgeted, and what we had received. And it really hits a lot of these hospitals especially the big hospitals. Middle‐level manager, County D

#### 3.1.4 Reduced financial autonomy

Resource mobilization (SPA 3) was also undermined by limited financial autonomy. First, in keeping with the requirements of the PFMA, all revenues raised by or received on behalf of the county government were transferred into one county revenue fund for public service provision.^14^, ^24^ In the past, public health providers were allowed to keep user fees revenues in their own bank accounts and draw on them through the authorization to incur expenditure. They therefore felt that transferring user fees revenues into the county revenue fund undermined their authority over finances, which limited their purchasing decisions and power and, demotivated both the management and staff.

Since the inception of the counties, we have been treating the cost sharing money or the facility improvement fund just like any other revenue for the county. This has been a contentious issue. Why should a facility produce revenue and then put it in the common basket? It has killed the morale of those people that collect the revenue and the morale of the practitioners and of course it has affected the general welfare of this community. Senior‐level manager, County H

Second, the county department of finance was the chief custodian of the budgeting process. However, limited consultations between the CDOH and the finance department hindered adequate planning and budgeting. This undermined purchasing through poor prioritization of needs for the health department and restricted access to financial resources.

The department of health has not been consulted whatsoever on what are their interests and priorities are. In a lot of the counties, this one included, the department of finance tends to do everything in the budget and more or less dictate terms to the other departments. Senior‐level manager, County C

#### 3.1.5 Weak accountability of the CDOH

Governments are expected to ensure accountability of purchasers (SPA 4). However, there was poor accountability of the CDOH. All county departments were required to demonstrate performance and financial accountability to the county government. For performance accountability, the CDOH were expected to align their annual development activities with the CIDP that outline the county's goals, implementation plans, and performance indicators for a 5‐year period.^24^, ^28^ The CDOH were also required to work towards attaining the national health performance indicators on service delivery and provide reports on the same.^22^ However, the CDOH did not adhere to these performance requirements with no repercussions reported.

We were mandated to do departmental reports on a quarterly basis to the county government which included what we had planned to do based on our work plan. However, we have not shared it with them. Senior‐level manager, County C

For financial accountability, the CDOH were answerable to the county treasuries and the general public for their use of public finances. The latter were also represented by the county assembly and constitutional bodies such as the Controller of Budget and the Office of the Auditor General. For example, the PFMA required the CDOH to develop a departmental sectoral plan in keeping with the CIDP that would provide a basis for budgeting.^24^ Failure to comply with the sectoral plans resulted in non disbursement of funds by the county treasury.

Our sanction is very simple because it is our office that approves. If money has been or had been used unlawfully then the sanction is you don't get approval. Senior‐level manager, County I

However, the county assemblies lacked the technical capacity to monitor the performance of the CDOH that would better inform the budgeting process. They were more focused on obtaining political mileage through funding of development expenditure, which yielded tangible results such as buildings.

A Member of the County assembly will ask, “That dispensary you were building, why has roofing not been done?” But those are not the key issues. The key issues are in the health outcomes. That is what shows whether the budget is actually working. Senior‐level manager, County E

### 3.2 CDOH—health care provider relationship

#### 3.2.1 Inadequate implementation of quality assurance mechanisms

Several SPAs relate to quality assurance in service delivery. One, a strategic purchaser should accredit providers taking into consideration the range and quality of services provided (SPA 5) and establish service contracts to this end (SPA 6). However, SPAs 5 and 6 were not met for two reasons. First, given that the CDOH owned public health care providers and had the responsibility of funding and equipping them (ie, there was a lack of purchaser provider split), they could not choose not to purchase services from a public health care provider on account of performance or quality (SPA 5). Second, the nature of the public integrated system where the CDOH and the health care facilities were not organizationally separate meant that there were no contracts and/or service agreements between the two (SPA 6).

Two, a strategic purchaser should develop formularies and treatment guidelines for service delivery (SPA 7). There were clear treatment guidelines for common illnesses and essential drug lists to standardize service delivery and guide commodity management across the tiers of health care, respectively.^29^, ^30^, ^31^, ^32^ All providers were expected to use the standard treatment guidelines and essential drug lists towards quality improvement in service provision.^33^ However, standard treatment guidelines and essential drug lists were often not available at health facilities, and where available, they were not often adhered to. In addition, there was limited evidence of the existence of therapeutic committees and quality improvement teams that would develop and oversee the implementation of these tools.

The health centers do not have MTCs [Medicine and Therapeutics Committees]. So now it will be the hospitals to guide the health centers and dispensaries. Middle‐level manager, County B

Third, a strategic purchaser should monitor provider performance and act on poor performance (SPA 11). There was provision for the CDOH to monitor provider performance through supervision of health care providers. This was to be done quarterly by county and subcounty health management teams as well as quality improvement teams. These teams assessed the quality of care through inspections, monitoring of health indicators, and monitoring of adherence to standard treatment guidelines and operating procedures. However, supervision and hence provider performance monitoring was not performed regularly due to inadequate or no funding for such activities that were not considered important by the treasury.

When we do budget, there are some things we hide like supervision. Even just supervision money, those ones you hide them under things like travel, accommodation and fuel … I hide them under there without saying supervision. Because if they [county treasury] see supervision, it will be out. Senior‐level manager, County C

Monitoring of provider performance was also limited by the lack of clear frameworks and reporting structures for monitoring and evaluating provider performance.

We had a very nice system where before our role was actually to inspect the facilities and fill the appropriate forms and send them to relevant bodies. Now that role has been put by the constitution to the counties yet there are no proper mechanisms. Senior‐level manager, County G

#### 3.2.2 Inadequate and untimely provider payment mechanisms

Purchasers are expected to design, implement, and modify provider payment methods to encourage efficiency and service quality (SPA 8). Related to design of provider payment methods (SPA 8), purchasers are expected to set provider payment rates that are adequate (SPA 9). The CDOH allocated line‐item budgets and salaries to public providers and their staff, respectively, for health service delivery.^14^, ^28^ Line‐item budgets mainly considered past patterns of expenditure. Public providers considered the line‐item budgets from the CDOH insufficient to meet service delivery. This led them to ration services and charge user fees for otherwise free services to enable sustainability of services.

This little budget that we have is for non‐pharmaceuticals. So, patients are asked to pay. Sometimes people pay even for the things they should not pay for. Senior‐level manager, County E

Staff salaries were fixed according to one's job group and not linked to performance. These were also considered insufficient, which was further exacerbated by delays in promotions that would have otherwise offered an increase in the salaries. This led to health worker demotivation and industrial actions that undermined the quality of health services delivered.

Salary is the reason why you see people going on strike everywhere. Because since we devolved people have not been promoted. So, no salary increments and no development. Middle‐level manager, County F

Purchasers are also expected to pay providers regularly (SPA 14). The delays in disbursement of funds to the CDOH highlighted under the section on government‐CDOH relationships had a knock‐on effect on disbursement of line‐item budgets to health care facilities and payment of staff salaries.

#### 3.2.3 Weak health management information systems

Purchasers are expected to secure information on services provided (SPA 10) and develop, manage, and use information systems (SPA 18). The CDOH required all health care providers to submit all health‐related information that emerged from their activities to enable evidence‐based planning and decision making.^15^ Information and communication technologies were expected to facilitate how health information was shared and accessed to enable delivery of quality and effective health care.^34^ In practice, health‐related information was provided through paper‐based registers and the Kenya Health Information System (an electronic information system). However, there was limited access to the electronic information system. Health care providers lacked computers, and where computers were available, regular network failures hindered the use of the electronic system. This undermined reporting and generation of health information that would otherwise have been used to monitor provider performance and support planning and decision making.

The partners brought us so many computers, they wired the whole place, but it is still not operational because of network failure every now and then. Facility‐level manager, County A

The use of health‐related information to support planning and decision making was undermined by poor quality of data. This was associated with shortages of health records information officers who were responsible for recording and reporting health information and the limited capacity of the CDOH to conduct regular support supervision and data quality checks. Multiplicity of reporting requirements including those for vertical programs also undermined quality by causing a strain on the system and its resources.

What is an issue, is the quality of those reports … When it comes to reporting, they designate one person now to combine all the registers. Senior‐level manager, County C

#### 3.2.4 Weak financial audit systems

A strategic purchaser should audit provider claims (SPA 12) and protect against fraud and corruption (SPA 13). The PFMA requires county governments to have internal and external audit arrangements with the aim of promoting transparency and accountability.^24^ In the CDOH purchasing arrangement, health care providers did not submit claims for reimbursement. Instead, they submitted financial statements in keeping with the budget policy statements approved by the county treasury. Some respondents indicate that audits were to be conducted quarterly, but this was not often done. In addition, there were few reported cases of sanctions applied to those who did not adhere to the financing requirements. Poor auditing was thought to have contributed towards the increasing reports of corruption in the health department.

I think is just fear of being audited and the fear that something might have gone wrong which they do not want uncovered. Because if you tell them we want to analyze your budgets and give you feedback on that, they ask you what it is that you want to dig out in their budgets which they do not know about. It is just that corruption issue than that whole cycle that creates the fear of being audited or being accountable to the people. Civil society FGD, County D

### 3.3 Citizen‐CDOH relationship

#### 3.3.1 Inadequate assessment of the service needs and preferences of the citizens

Purchasers are expected to assess the service needs, preferences, and values of the citizens and use these to design service benefits/entitlements (SPA 19) and inform the citizens of their entitlements and obligations (SPA 20). The KEPH services offered in the county‐owned public hospitals were anchored on the disease burden in Kenya, cultural acceptability, equity, cost‐effectiveness, and value for money.^25^ The CDOH was also constitutionally mandated to facilitate public participation through which it would engage the public, represent their interests, and deliver services that respond to their needs.^14^ While county respondents (senior and middle level managers) reported holding public participation meetings, members of the public reported that such meetings on health were not held. Where they were held, the forums were not well publicized, which limited the public's knowledge of the presence of and attendance to such meetings. The forums were also held in distant venues, thereby limiting public attendance due to geographical and financial barriers. Lastly, the forums were not inclusive, thereby limiting input from marginalized members of the community.

In public participation forums, you will find very few people and these are the people who have the information. But the grass root, the community people, are never represented in some of these forums so their views don't reach the county government. Civil society FGD, County I

#### 3.3.2 Inadequate delivery of service entitlements

Purchasers are expected to ensure citizens can access their entitlements (SPA 21). The public was entitled to primary and secondary health care services from the county owned providers. These services are composed of preventive, promotive, curative, and rehabilitative health services for communicable and noncommunicable diseases in Kenya.^25^ In practice, however, none of the public health care providers offered the comprehensive range of health services due to financial, infrastructure, and human resource constraints.

There is no facility that offers the comprehensive range of services as outlined in the KEPH even the hospital here. There are certain services that are outlined there that we cannot provide due to financial and human resources constraints. Senior‐level manager, County C

Citizen's access to service entitlements was also impaired by financial barriers. While citizens could access free primary care (thanks to user fee removal at primary care level), access to secondary care was limited by out‐of‐pocket (OOP) payments. In fact, there were reports of some counties raising the OOP payments even higher in order to generate more revenue, which limited access even further.

We sent her to the center but do you know that woman just went home and slept … “Ah! I don't have money” She's basically now waiting to die. The average person here does not have money to go to facility X [secondary care provider] for treatment, they don't. Female FGD, County D

Some of the proposed hospital charges are higher than the current because we also have targets as a department. We are given targets that we are to raise 100 million internally. It's up to us now to find out how we raise that 100 million. You may raise them [hospital charges] to a point where people just stop seeking services here. Senior‐level manager, County C

In addition to the unaffordable costs, nonfunctional waiver mechanisms further limited access to needed secondary care. Waiver mechanisms existed for those without the ability to pay. However, they suffered from political interference, whereby people with ability to pay were exempted from making payments due to political affiliations while those with no ability to pay and no political affiliation were forced to pay. Also, waivers only applied to those services that were received from the public health facility itself. This meant that in case services or health supplies were unavailable in public facilities, users incurred OOP costs.

People who could obviously have paid, you are called and told, “Let that one go!” It's really unfair because you will see people who actually needed to be waived, who needed help but they do not really get the help that those with political links get. Facility‐level manager, County B

#### 3.3.3 Ineffective complaints and feedback mechanisms

Purchasers are also expected to establish effective complaints and feedback mechanisms (SPA 22). Mechanisms for providing complaints and feedback included suggestion boxes; direct communication with facility managers, county managers, members of the county assembly, and civil society organizations; and social media platforms such as county websites and Facebook pages. However, members of the public felt that these mechanisms did not assure anonymity or confidentiality, which limited their use for fear of victimization.

Many people are afraid of reporting because when you report an incident, they mark you and you become [a target]. Mixed FGD, County A

In other instances, members of the public were not aware of the existence of these complaints and feedback mechanisms. Lack of clear complaints and feedback structures caused frustration among the citizens, which limited their use and capacity to bring about change. This undermined answerability of providers to the citizens.

I went to the pharmacy and they sold for me a bandage that was written, “Not for sale.” After they had used it, I kept the wrapper and I went back to ask about this incident but the pharmacist referred me to the matron. The matron said that the one who is in charge is the administrator. I went all around until I decided to give up. Male FGD, County D

While some health managers reported using the complaints and feedbacks provided to improve service delivery, others reported not having the capacity to address the complaints raised. This limited the effectiveness of the mechanisms.

They [citizens] want better quality services at lower prices. They believe they were shortchanged and that they were promised things they have not gotten. So, they come to complain to you on the ground, but you don't have any power to effect anything. All you can do is listen and forward it. Facility‐level manager, County G

#### 3.3.4 Poor financial accountability of the CDOH to citizens

Purchasers are expected to publicly report on the use of resources and other measures of performance (SPA 23). The CDOH were required to uphold openness in public finance through public participation and clear fiscal reporting.^14^ While county managers reported involving the public in budget making and budget dissemination forums, members of the public reported not being involved. They also had no access to the budget documents, financial statements, or implementation reports. Where access was available, the technical content of some of the reports made it difficult for members of the public to adequately judge the CDOH's fiscal performance. This undermined purchasing by limiting transparency and accountability of the purchasers to the citizens.

Public participation especially on the budget is the first avenue that the county government can engage the community on accountability issues but this really does not happen. The other challenge that we have is the issue of accessing those major budgetary documents. We need to see the budget review paper, we need to see the budget estimates but that information is so difficult to find. Civil Society FGD, County I

We noted that for the public, some of these financial reports are too technical. So, we are trying to simplify them in such a way that they are able to understand. Senior‐level manager, County C

## 4 DISCUSSION

The assessment of the purchasing arrangement within the county integrated public system in Kenya shows that the majority of the 23 SPAs along the three purchaser relationships were not met. With regard to SPAs on the government‐purchaser axis, the overarching finding is that the government's (national and county) role as a steward for the purchasing function of the CDOH was weak. Good stewardship in purchasing involves development of policies that support purchasing and effective regulation to enable providers achieve the stated objectives.^9^ While on paper the national and county governments provided direction for purchasing through laws and policies, enforcement mechanisms were weak. This was further compounded by inadequate budgetary allocations for service delivery by the government (national and county). Insufficient resource allocation by the government is a barrier to good stewardship in strategic purchasing, as it goes against its obligations of financing health services and incentivizes opportunistic behavior by the providers^9^ as seen in our study.

Along the purchaser‐provider axis, the fact that purchasers and providers are not organizationally separate in a public integrated system compromised strategic purchasing in two ways. First, the CDOH could not leverage their purchaser role to assure quality by only purchasing services from those providers that met a defined quality standard. Provider selection is an integral aspect of strategic purchasing that allows purchasers to match interventions to providers' ability to deliver good quality and timely services at the lowest possible cost.^35^ Where provider selection is not possible, clear systems for monitoring provider performance are needed for quality improvement.^8^, ^10^ For an integrated system, this can be achieved through a robust health information system,^8^, ^35^, ^36^ proper financial and risk management system,^8^, ^35^ and timely information sharing between purchasers and providers.^4^ The county health information system lacked these key features, thus undermining strategic purchasing. Second, the CDOH had limited financial autonomy, which limited its ability to respond to service needs and requirements. Purchaser‐provider split in a publicly funded system could create autonomy of the providers and minimize the direct influence of the government on the purchasing decisions,^37^ increase efficiency,^9^ and improve performance and accountability.^38^

Provider payments through line‐item budgets and staff salaries were passive forms of purchasing that did not incentivize providers to improve quality of care or efficiency of service delivery. Similar findings were observed in the Nigerian and South African public integrated systems where these payments methods were in use.^39^, ^40^ Line‐item budgets limit flexibility of resource allocation by purchasers and flexibility of resource use by providers^4^ and are weakly aligned with the services provided in the benefit package.^41^ Salaries have been shown to undermine health worker productivity, motivation, and quality of care.^42^

The citizen‐purchaser axis was characterized by poor public participation mechanisms, which limited public accountability and responsiveness of the CDOH to the needs of the people. This was exacerbated by weak complaints and feedback mechanisms. Similar weaknesses were reported in the Nigerian and South African integrated systems.^39^, ^40^

Drawing from these findings, we make several recommendations. One, county governments should strengthen the accountability mechanisms between themselves and the county health departments through, for example, regular monitoring of and linking financing to performance. Such an approach will ensure that the CDOH are accountable not only for resources but also for service delivery. Two, both the national and county governments should increase the funding allocation to the MOH and CDOH, respectively. Three, county governments should review their by‐laws to provide increased financial autonomy and flexibility to the CDOH that will enhance efficiency. Four, related to autonomy, the national and county governments should review the legal framework to introduce elements of purchaser‐provider split between the CDOH and public health care providers. Under this arrangement, the relationship between the CDOH and health care facilities could be contractual with clear standards of performance along a range of domains that include financial, service delivery, and quality. While we appreciate that experience with purchaser provider‐split varies across context and is dependent on how it is designed and implemented,^43^, ^44^, ^45^, ^46^ its introduction has the potential to enhance a purchaser's capacity to enforce accountability of providers.^47^ Five, county governments should assess the service delivery infrastructure needs of the CDOH and make appropriate investments to strengthen service delivery. In doing this, county governments should explore possibilities of intercounty collaboration to leverage on shared resources particularly for expensive medical equipment and highly specialized health care personnel. Six, the national and county governments should review provider payment mechanisms with the aim of introducing elements that would incentivize efficiency and quality. This could include shifting from line‐item budgets to global budgets initially, and then progressively considering other payment mechanisms such as capitation for primary health care services. It could also include moving from fixed salaries for all health care workers to hybrid systems that include fee for service for specialist services. Seven, county governments should strengthen the health management information system and incentivize providers to submit timely and good quality information. This can be done through financial incentives or sanctions for compliance or noncompliance, respectively. Lastly, county governments should strengthen public participation and complaints and feedback mechanisms by incorporating these as part of monitoring and evaluation of provider and purchaser performance requirements.

## 5 CONCLUSION

Our findings show that while there are frameworks that could promote strategic purchasing of the CDOH, strategic purchasing is impaired by poor implementation of these frameworks and the inherent weaknesses of a public integrated purchasing system that lacks purchaser‐provider split. Given that the CDOH is the largest purchaser of health care services in Kenya, ensuring that its purchasing actions are strategic should be prioritized. This requires formulating and implementing reforms to improve the purchasing function of the CDOH, as purchasing reforms have the potential to accelerate the country's progress towards the attainment of UHC. A limitation of the study is the inability to statistically generalize the findings to the whole of Kenya or other settings outside of Kenya. However, in keeping with the intentions of qualitative health system research, the intention is to achieve analytical generalizability (ie, generalizing to theory) rather than statistical generalizability. The study also provides useful insights into the purchasing arrangements of the CDOH that can be considered and tested in other Kenyan counties, and other comparable settings.

## CONFLICT OF INTEREST

The authors have no competing interests.

## AUTHOR CONTRIBUTIONS

J.C. conceptualized the study; K.M., P.N., and S.M. collected data. K.M., P.N., and R.M. performed the initial analysis. All authors contributed to further analysis. R.M. wrote the first draft of the manuscript. All authors contributed to subsequent drafts.
